# Supplementary material for: Hydrophobic residues in S1 modulate enzymatic function and voltage sensing in voltage-sensing phosphatase
Source: J Gen Physiol. 2024 May 21;156(7):e202313467. doi: 10.1085/jgp.202313467 (PMC11109755; doi:10.1085/jgp.202313467)
Supplement: Table S1 — lists the used primers. [file JGP_202313467_TableS1.docx]

| Supplementary Table 1: Primers | | |
| --- | --- | --- |
| **His forward** | GGGGATCCGCCACCATGCATCATCACCACCACCATGAGGGATTCGAC  GGT TC |  |
| **His reverse** | GAACCGTCGAATCCCTCATGGTGGTGGTGATGATGCATGGTGGCGGA  TCCCC |  |
| **FLAG forward** | GGGGATCCGCCACCATGGACTACAAAGACGATGACGACAAGGAGGGA  TTCGACGGTTC |  |
| **FLAG reverse** | GAACCGTCGAATCCCTCCTTGTCGTCATCGTCTTTGTAGTCCATGGTGG  CGGATCCCC |  |
| **G214C forward** | GAAACAGGAGCCGATTGTTTGGGGAGATTG |  |
| **G214C reverse** | CAATCTCCCCAAACAATCGGCTCCTGTTTC |  |
| **C363S forward** | GATAGCGATTCACTCTAAAGGCGGGAAG |  |
| **C363S reverse** | CTTCCCGCCTTTAGAGTGAATCGCTATC |  |
| **F127A forward** | GGAGTCTTCCTAATTGCATTGGACATCATCCTCATG |  |
| **F127A reverse** | CATGAGGATGATGTCCAATGCAATTAGGAAGACTCC |  |
| **I131A forward** | CTAATTTTCTTGGACATCGCACTCATGATCATTGATC |  |
| **I131A reverse** | GATCAATGATCATGAGTGCGATGTCCAAGAAAATTAG |  |
| **I134A forward** | CATCATCCTCATGGCAATTGATCTCAGTC |  |
| **I134A reverse** | GACTGAGATCAATTGCCATGAGGATGATG |  |
| **L137A forward** | CTCATGATCATTGATGCCAGTCTTCCAGGAAAAAGTG |  |
| **L137A reverse** | CACTTTTTCCTGGAAGACTGGCATCAATGATCATGAG |  |
| **F127A I131A forward** | GTCTTCCTAATTGCATTGGACATCGCACTCATGATC |  |
| **F127A I131A reverse** | GATCATGAGTGCGATGTCCAATGCAATTAGGAAGAC |  |
| **I134A L137A forward** | CTCATGGCAATTGATGCCAGTCTTCCAGGAAAAAGTG |  |
| **I134A L137A reverse** | CACTTTTTCCTGGAAGACTGGCATCAATTGCCATGAG |  |
