# Supplementary material for: Hydrophobic residues in S1 modulate enzymatic function and voltage sensing in voltage-sensing phosphatase
Source: J Gen Physiol. 2024 May 21;156(7):e202313467. doi: 10.1085/jgp.202313467 (PMC11109755; doi:10.1085/jgp.202313467)
Supplement: Table S2 — lists VCF and the kinetics of S4 motions from 200 mV step. [file JGP_202313467_TableS2.docx]

| Supplementary Table 2: VCF, kinetics of S4 motions from 200 mV step | | | | | |
| --- | --- | --- | --- | --- | --- |
|  |  | **Activation** | | **Repolarization** | |
|  | **n** | **𝜏_a1_** | **𝜏_a2_** | **𝜏_r1_** | **𝜏_r2_** |
| **WT** | 10 | 0.018 ± 0.005 | 0.17 ± 0.01 | 0.053 ± 0.004 | N/A |
| **F127A** | 10 | 0.013 ± 0.002 | 0.12 ± 0.01 * | 0.0602 ± 0.006 | N/A |
| **I131A** | 13 | 0.015 ± 0.003 | 0.15 ± 0.02 | 0.069 ± 0.004 | N/A |
| **I134A** | 15 | 0.010 ± 0.001 | 0.15 ± 0.01 | 0.066 ± 0.004 | N/A |
| **L137A** | 10 | 0.010 ± 0.001 | 0.12 ± 0.01 * | 0.058 ± 0.004 | 0.24 ± 0.03 |
| **S1Q** | 10 | 0.007 ± 0.001 * | 0.11 ± 0.02 * | 0.06 ± 0.01 | 0.30 ± 0.05 |

* Student’s t-test p < 0.05
