# Supplementary material for: Hydrophobic residues in S1 modulate enzymatic function and voltage sensing in voltage-sensing phosphatase
Source: J Gen Physiol. 2024 May 21;156(7):e202313467. doi: 10.1085/jgp.202313467 (PMC11109755; doi:10.1085/jgp.202313467)
Supplement: SourceData F6 — is the source file for Fig. 6. [file JGP_202313467_SourceDataF6.pdf]

F127A and L137A western blots from Figure 6A

Sample input

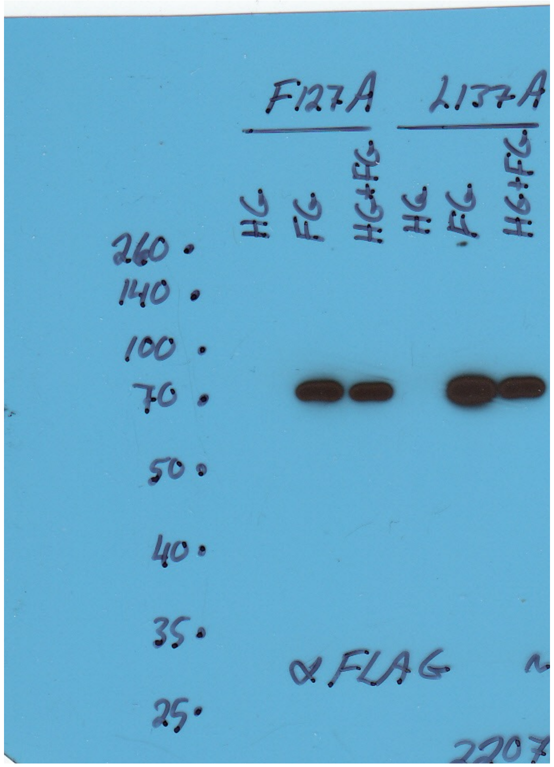

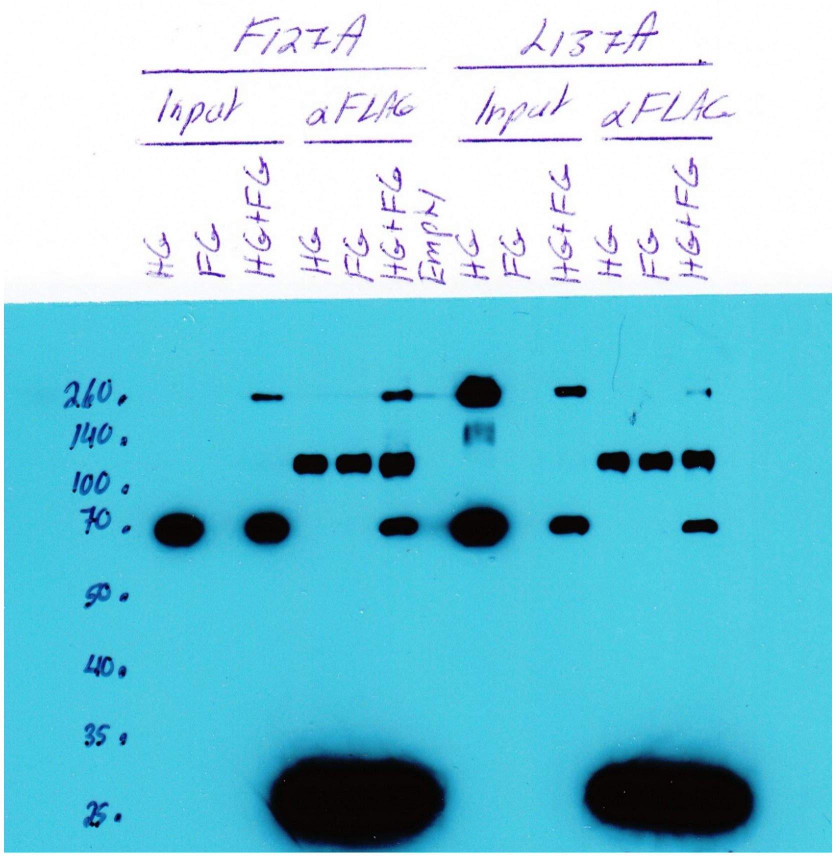

IB: Anti-FLAG

IB: Anti-His

I131A, I134A and S1-Q (top) western blots from Figure 6

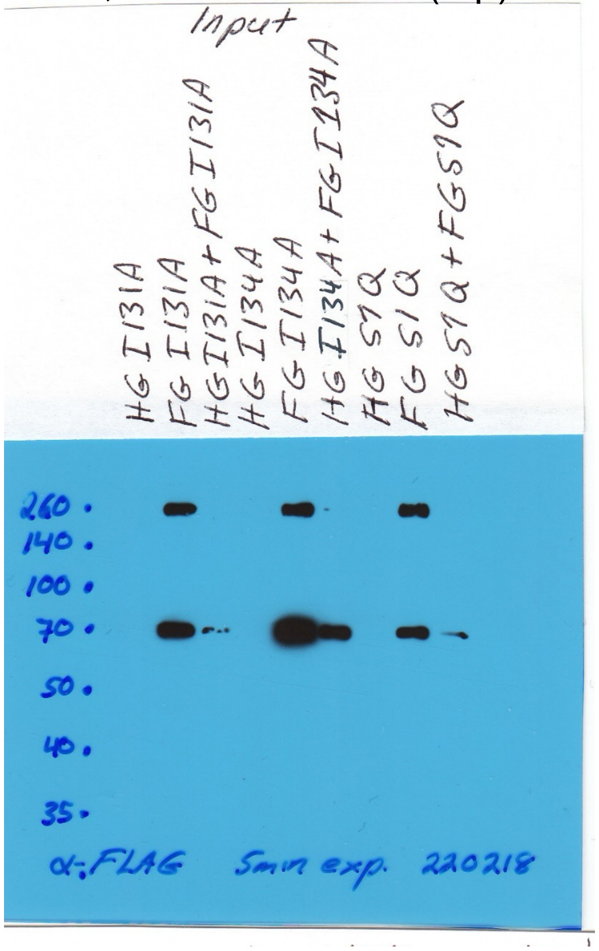

IB: Anti-FLAG

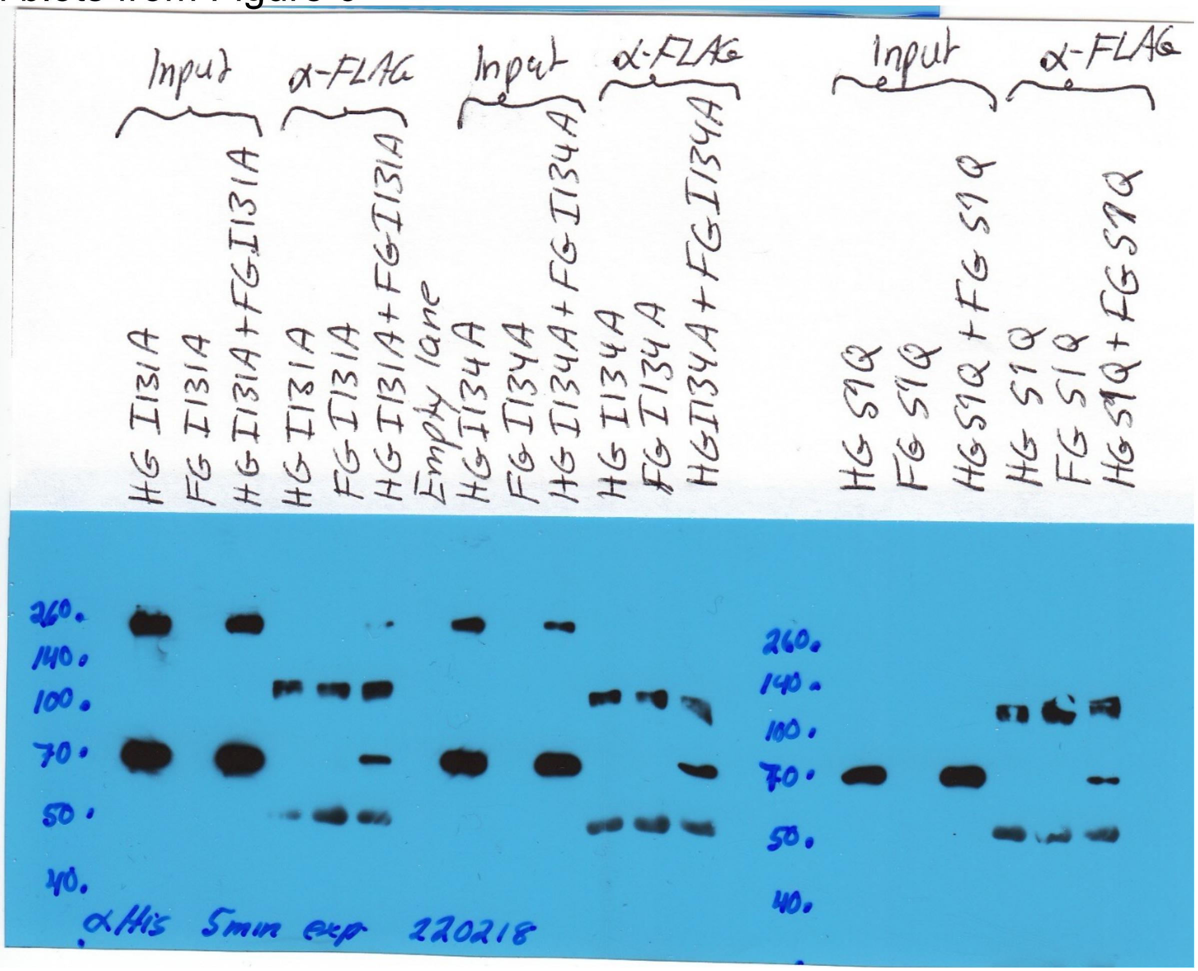

IB: Anti-His

S1-Q (bottom) western blot from Figure 6B

Sample input

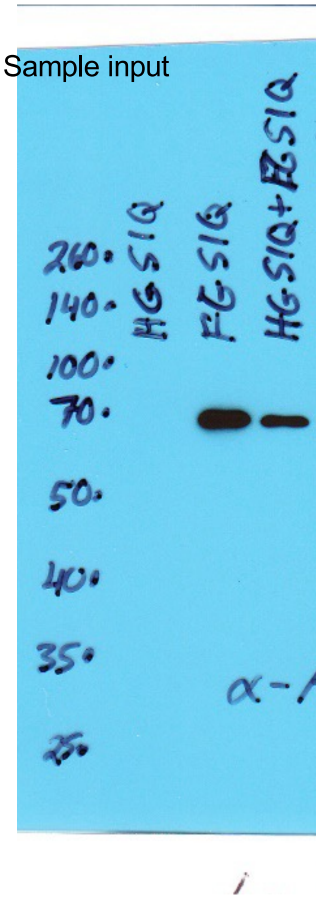

IB: Anti-FLAG

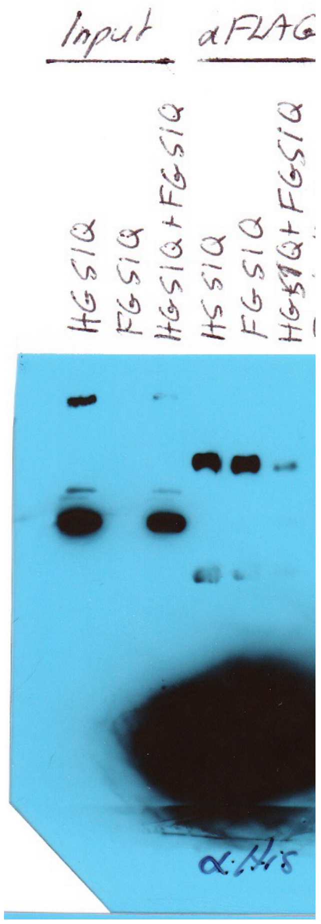

IB: Anti-His
